# HCRP-1 regulates cell migration and invasion via EGFR-ERK mediated up-regulation of MMP-2 with prognostic significance in human renal cell carcinoma

**DOI:** 10.1038/srep13470

**Published:** 2015-08-25

**Authors:** Feifei Chen, Junpeng Deng, Xin Liu, Wang Li, Junnian Zheng

**Affiliations:** 1 Jiangsu Center for the Collaboration and Innovation of Cancer Biotherapy, Cancer Institute, Xuzhou Medical College, Xuzhou, Jiangsu, China; 2 The Affiliated Hospital of Xuzhou Medical College, Xuzhou, Jiangsu, China; 3 Department of Urology, Suzhou Municipal Hospital, Suzhou, China

## Abstract

Previous studies indicated a role of hepatocellular carcinoma-related protein-1(HCRP-1) in human cancers, however, its expression pattern in renal cell carcinoma (RCC) and the molecular mechanism of HCRP-1 on cancer progression have not been characterized. In the present study, HCRP-1 expression was examined in a RCC tissue microarray. The negative expression of HCRP-1 was significantly correlated with tumor grade (*P* = 0.002), TNM stage (*P* = 0.001) and pT status (*P* = 0.003). Furthermore, we showed a strong correlation between negative HCRP-1 expression and worse overall and disease-specific survival (*P* = 0.0003 and *P* = 0.0012, respectively). Knockdown of HCRP-1 promoted cell migration and invasion in 786-O and OS-RC-2 cell lines. HCRP-1 depletion increased matrix metalloproteinase (MMP)-2 protein level, with increased extracellular signal-regulatedkinase (ERK) phosphorylation, which could be reversed by ERK siRNA or ERK inhibitor, PD98059. Further analysis showed that HCRP-1 knockdown induced epidermal growth factor receptor (EGFR) phosphorylation. Treatment with EGFR inhibitor or EGFR siRNA blocked HCRP-1-mediated up-regulation of EGFR, ERK phosphorylation and MMP-2 expression. In summary, our results showed that negative HCRP-1 expression is an independent prognostic factor for RCC patients and promotes migration and invasion by EGFR-ERK-mediated up-regulation of MMP-2. HCRP-1 may serve as a therapeutic target for RCC.

RCC, the third most common urologic cancer, is a very aggressive disease that accounts for about 3% of all malignancies and 90% to 95% of kidney neoplasm^1^,^2^. Systemic treatments have been made in the management of RCC over the past few years, but 30% of patients develop metastatic disease^3^, and median survival of those patients is only about 13 months^4^. Therefore, there is a significant need for us to find appropriate biomarkers, which are crucial to the prediction, therapy and evaluation of prognosis of RCC patients.

EGFR is a transmembrane receptor tyrosine kinase (RTK) that plays an essential role in governing multiple cellular processes including cell proliferation, survival, and cell migration^5^,^6^. Hyperactivation of EGFR-dependent signal transduction usually accompanies tumor development, and cancer patients with unbalanced activation of EGFR generally present with a more aggressive disease leading to unfavorable clinical prognosis^7^. Therefore, targeting of EGFR as well as its downstream pathways is a mainstay of treatment in many malignant diseases, including RCC. The human endosomal sorting complex required for transport-1 (ESCRT-I) is essential for the binding and sorting of ubiquitinated transmembrane proteins including EGFR into internal vesicles of multivesicular bodies (MVBs) and subsequent degradation with the lysosome^8^. It consists of the four subunits, multivesicular body sorting factor 12, tumor susceptibility gene 101, vacuolar protein sorting 28 homologue and HCRP-1^9^,^10^.

Among them, HCRP-1, also known as vacuolar protein sorting 37 homologue A (hVps37A), is located on the short arm of chromosome 8^11^. For this region, 8p22, loss of heterozygosity (LOH) occurs to a high frequency in several human cancers including RCC^12^. HCRP-1 was first identified abundant in normal human liver tissue but significantly reduced or undetected in hepatocellular cell carcinoma (HCC) tissues^13^. In a recent study of ovarian cancer, it was reported that HCRP-1 down-regulated expression is associated with activation of EGFR, and its expression has a significant impact on the prognostic value of EGFR expression. Silencing HCRP-1 induces invasive phenotype *in vitro* and tumor growth *in vivo*^14^. Moreover, low HCRP-1 expression is proved to be of adverse prognostic significance in patients with oropharyngeal squamous cell carcinoma (OOSCC) who received preoperative chemoradiotherapy^15^. These studies indicate that HCRP-1 plays an important role in cancer progression with function as a tumor suppressor. Nonetheless, HCRP-1 has been poorly characterized in RCC so far.

In the present study, we evaluated HCRP-1 staining in RCC tissues and paired non-cancerous tissues using tissue microarray technology and analyzed the correlation between HCRP-1 expression and clinicopathologic features. Furthermore, we showed, for the first time to our knowledge, a role for HCRP-1 in suppression of migration and invasion in human RCC cells. Finally, we provided evidence that HCRP-1-knockdown up-regulated MMP-2 expression through the EGFR-ERK signaling pathway in this process.

## Material and Methods

### Patients and samples

A renal cell carcinoma TMA was purchased from Shanghai Xinchao Biotechnology (Shanghai, China). Tumors were staged according to the 2010 revised TNM system as follows^16^: 76 cases with stages I-II and 14 cases with stages III-IV. Histologicalgrades of tumors were defined according to the WHO criteria as follows: 73 cases of low grade (Grade I and II), 17 cases of high grade (Grade III and IV). In addition, it includes 90 cases of tumor adjacent normal renal tissue. Follow-up information was obtained by reviewing patient medical records.

### Antibodies and Reagents

Anti-HCRP-1 rabbit polyclonal antibodies were purchased from proteintech (Wuhan, China). Rabbit monoclonal antibodies to MMP-2, MMP-9, p38, phospho-p38, C-Jun amino terminal kinase (JNK), phospho-JNK, MEK1/2, phospho-MEK1/2, c-Raf, phospho-c-Raf, Ras and phospho-Ras were purchased from Cell Signaling Technology (Beverly, MA). Antibodies against EGFR, phospho-EGFR (Tyr1173), ERK and phospho-ERK were from Santa Cruze (CA, USA). Mouse anti-β-actin was from Boster Biotechnology (Wuhan, China). ERK1/2 inhibitor, PD98059 and EGFR inhibitor, AG1478 were from Abcam (Shanghai, China).

### Immunohistochemistry of TMA

Immunohistochemistry staining was performed as described previously^17^. The TMA slide was incubated with HCRP-1 antibody (1:25) overnight at 4 °C, and diaminobenzidine (DAB; Zhongshan Biotech, Beijing, China) was used to produce a brown precipitate. For the TMA staining evaluation, the immunoreactivity was assessed blindly by two independent observers using light microscopy (Olympus BX-51 light microscope), and the image was collected by Camedia Master C-3040 digital camera. The expression of HCRP-1 was graded as positive when 10% of tumor cells showed immunopositivity. Biopsies with 10% tumor cells showing immunostaining were considered negative^18^.

### Cell Culture

Human RCC cell lines 786-O and OS-RC-2 were purchased from the Shanghai Institute of Biochemistry and Cell Biology, Chinese Academy of Sciences (Shanghai, China). 786-O and OS-RC-2 cells were cultured in RPMI1640 medium supplemented with 10% fetal calf serum (Invitrogen, Shanghai, China). Cells were in a 37 °C humidified incubator with 95% air, 5% CO_2_.

### siRNA transfection

Non-specific control siRNA or HCRP-1 siRNA, and siRNAs for ERK1/2 and EGFR were purchased from Integrated Biotech Solutions (Shanghai, China). The sequences of siRNAs are as follows:HCRP-1, 5′-GACACUGUUUCUUCUUCAACA-3′; ERK1, 5′-GACCGGAUGUUAACCUUUATT-3′; ERK2, 5′-CCAAAGCUCUGGACUUAUUTT-3′; EGFR, 5′-CACAGUGGAGCGAAUUCCUTT-3′. The cells were transfected with siRNA using siLentFect Lipid Reagent (Bio-Rad, Hercules, CA, USA) according to the manufacturer’s instructions.

### Migration assay

Cell migration was determined by using a modified two chamber migration assay with a pore size of 8 μm. For migration assay, 3 × 10^4^ 786-O and OS-RC-2 cells were seeded in serum-free medium in the upper chamber. After 12 h incubation at 37 °C, cells in the upper chamber were carefully removed with a cotton swab and the cells that had traversed the membrane were fixed in methanol and stained with leucocrystal violet. The number of migration cells was determined by counting the leucocrystal violet stained cells. For quantification, cells were counted under a microscope in five fields (up, down, median, left, right, ×200).

### Invasion assay

The invasion assay was performed using a modified two chamber plates with a pore size of 8 μm. The transwell filter inserts were coated with matrigel (BD Biosciences, NJ, USA). 5 × 10^4^ 786-O and OS-RC-2 cells were seeded in serum free medium in the upper chamber. After 24 h incubation at 37 °C, noninvasive cells were gently removed from the top of the matrigel with a cotton-tipped swab. Invasive cells at the bottom of the matrigel were fixed in methanol, stained with leucocrystal violet and counted.

### Western blot analysis

For cancer cells, forty-eight hours (siRNA) after transfection, cells were harvested from the plates. Then aliquots of cell extracts were separated on a 10% SDS-polyacrylamide gel. The proteins were then transferred to nitrocellulose membrane and incubated overnight at 4 °C with appropriate primary antibodies. After incubation with peroxidase-coupled anti-mouse or anti-rabbit IgG at 37 °C for 2 hours, membranes were then washed and scanned on the Odyssey Two-Color Infrared Imaging System (LI-COR Biotechnology, Lincoln, Nebraska, USA). Each western blot was repeated three times.

### Statistical analysis

SPSS version 13.0 for Windows (SPSS Inc., Chicago, IL) was used for all analyses. Data are expressed as the means ± SD. Two-factor analysis of variance procedures and the Dunnett’s t test were used to assess differences within treatment groups. For TMA, the association between HCRP-1 staining and the clinicopathologic parameters of the RCC patients were evaluated by χ^2^ test. Difference between each patient’s tumor with its normal counterpart was evaluated by paired χ^2^ test. The Kaplan-Meier method and log-rank test were used to evaluate the correlation between HCRP-1 expression and patient survival. Hazard ratios (HR) and 95% confidence intervals (CI) were calculated using multivariate Cox proportionalhazards regression models to analyze the independent impact of clinicopathologic factors and HCRP-1 on survival. A *P*-value equal to or <0.05 was considered statistically significant.

## Results

### HCRP-1 expression is decreased in human RCC

**Figure 1:**
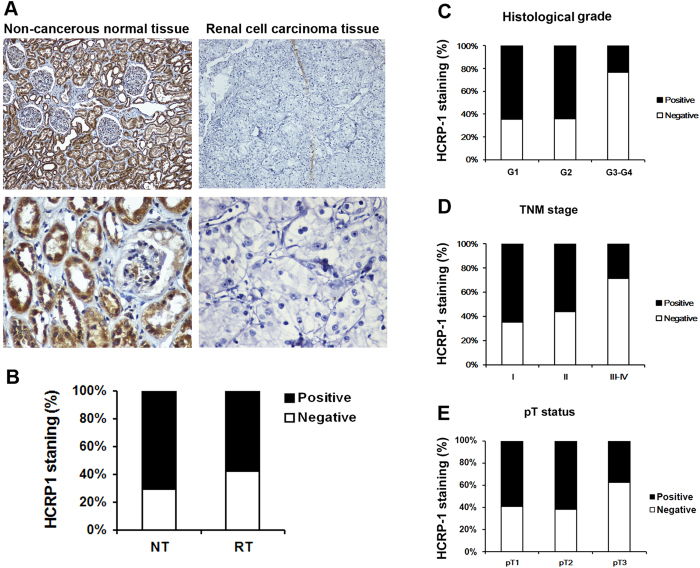
Correlation between HCRP-1 expression and clinicopathologic parameters in RCC. (**A**) Representative immunohistochemical photographs were taken at different magnifications in tumor adjacent normal renal tissue and renal carcinoma tissues (Top panel ×100, bottom panel ×400). (**B**) Compared with that in the tumor adjacent normal renal tissue, the overall expression level of HCRP-1 in the renal cell carcinoma tissues was significantly lower (*P* < 0.01, χ^2^ test). (**C**) Decreased HCRP-1 expression was correlated with histological grade (*P* < 0.005, χ2 test, comparing I-II versus III-IV). (**D**) Decreased HCRP-1 expression was correlated with TNM stage (*P* < 0.005, χ2 test, comparing I-II versus III-IV). (**E**) Decreased HCRP-1 expression was correlated with depth of invasion (*P* < 0.005, χ2 test, comparing pT1 versus pT2–pT3).

We first determined whether HCRP-1 expression is changed in human RCC. Immunohistochemistry staining was performed in TMA slide containing RCC tissues and paired non-cancerous tissues. We found that HCRP-1 expression was localized in the cytoplasmic (Fig. 1A). In normal renal tissues, there was strong cytoplasmic immunostaining in normal renal tubule epithelia. Positive HCRP-1 staining was recorded in 71.1% (64 of 90 cases). Of the 90 patients with RCC, positive expression of HCRP-1 was observed in 42.2% (38 of 90 cases) (Fig. 1B). A significant lower expression of HCRP-1 was observed in the carcinoma tissues when compared with normal human renal tissues (*P* = 0.001, paired χ^2^ test).

### Correlation of HCRP-1 expression with clinicopathological parameters

The relationship between HCRP-1 expression and various clinicopathological features was investigated (Table 1). Our data showed that decreased expression of HCRP-1 showed a significant correlation with histological grade (comparing I-II versus III-IV) (*P* < 0.005, Fig. 1C). Because TNM stage is an important prognostic marker for patients with RCC, so we studied if HCRP-1 expression correlates with TNM stage. We found HCRP-1 staining was dramatically decreased in TNM stages III-IV compared with stages I-II (*P* < 0.005, Fig. 1D). We also found decreased HCRP-1 expression was significantly correlated with depth of invasion (comparing pT1 versus pT2-pT3) (*P* < 0.005, Fig. 1E). However, we did not find significant correlation between HCRP-1 expression with other clinicopathologic variables, including age, gender and tumor size.

### HCRP-1 expression and patients survival

**Figure 2:**
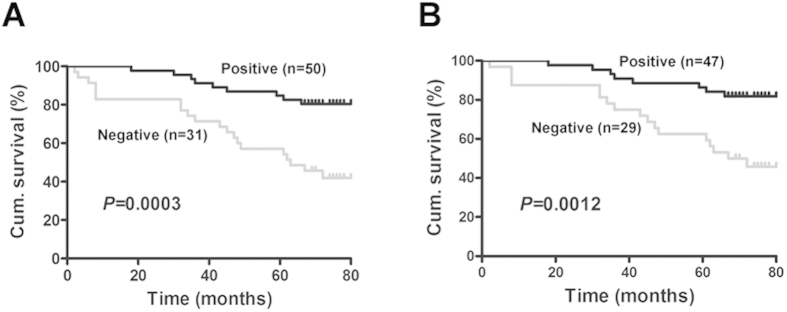
HCRP-1 expression is associated with patients’ survival. (**A**) Kaplan–Meier estimates of the probability of overall survival according to low and high HCRP-1 expression of 81 patients with RCC (*P* = 0.017). (**B**) Kaplan–Meier estimates of the probability of disease-specific survival according to low and high HCRP-1 expression of 78 patients with RCC (*P* = 0.028). Cum. indicates cumulative.

Overall survival and disease-specific survival were used for survival analysis. The disease-specific and overall mortality events were 76 and 81, respectively. The average length of the follow-up is 62.2. Kaplan-Meier survival analysis showed a significantly lower overall survival in patients with HCRP-1 negative RCC than those with positive HCRP-1 expression (*P* = 0.017, log rank test) (Fig. 2A). In addition, the negative HCRP-1 staining was correlated with worse disease-specific survival (*P* = 0.028, log rank test) (Fig. 2B).

Furthermore, we examined whether HCRP-1 expression is an independent prognostic marker for RCC. The independent impact of HCRP-1 expression on disease-specific survival and overall survival was assessed by Cox regression models adjusted for age, gender, tumor size, pathological grade and TNM stage (Table 2). In these analyses, low HCRP-1 expression remained significantly associated with shorter disease-specific survival (HR 0.379, 95% CI 0.163–0.882, *P* = 0.024) and shorter overall survival (HR 0.384, 95% CI 0.172–0.861, *P* = 0.020). Thus, low HCRP-1 expression is an independent poor prognostic factor in patients with RCC who received preoperative chemoradiotherapy.

### Loss of HCRP-1 promotes RCC cells migration and invasion *in vitro*

**Figure 3:**
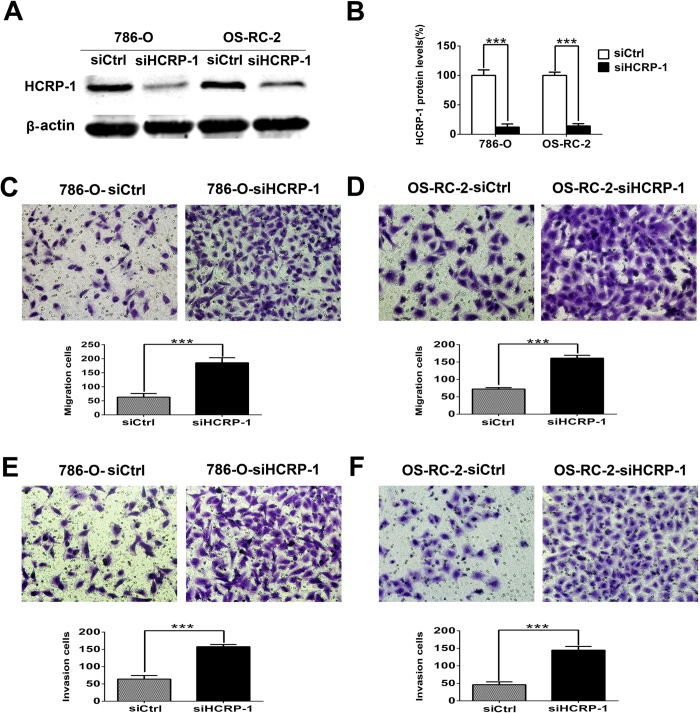
Effect of the reduction in HCRP-1 expression on the abilities of cell motility *in vitro*. (**A**,**B**) Forty-eight hours after transfection, the expression of HCRP-1 in the 786-O and OS-RC-2 cells was evaluated by western blotting. β-actin was used as an internal control. (**C**–**F**) Cell migration and Matrigel cell invasion assays were performed in 786-O and OS-RC-2 cells after transfection, respectively. Representative fields of migrating or invading cells on the membrane (magnification, ×200). The data are presented as mean ± SD for triplicate determinations. ***P < 0.001.

Because low HCRP-1 expression is associated with poor prognosis, supporting HCRP-1 may play an important role in one or more steps of tumor metastasis, we investigated the involvement of HCRP-1 in RCC cells migration and invasion. We failed to construct HCRP-1 plasmid to explore the effect of HCRP-1 on RCC cells migration and invasion. So, we transiently transfected 786-O and OS-RC-2 cells with control siRNA and HCRP-1 siRNA in this study, respectively. Forty-eight hours after transfection, HCRP-1 protein was significantly knockdown in cancer cells (Fig. 3A,B). Transfected cells were subjected to cell migration assay and invasion assay. In cell migration assay, we found that the ability of cell migration was drastically increased after decreased expression of HCRP-1 in both 786-O and OS-RC-2 cells (Fig. 3C,D). In cell invasion assay, the results were corresponded with the cell migration assay, respectively (Fig. 3E,F).

### Inhibition of HCRP-1 induces MMP-2 expression and activates the EGFR and MAPK-ERK signaling pathway

**Figure 4:**
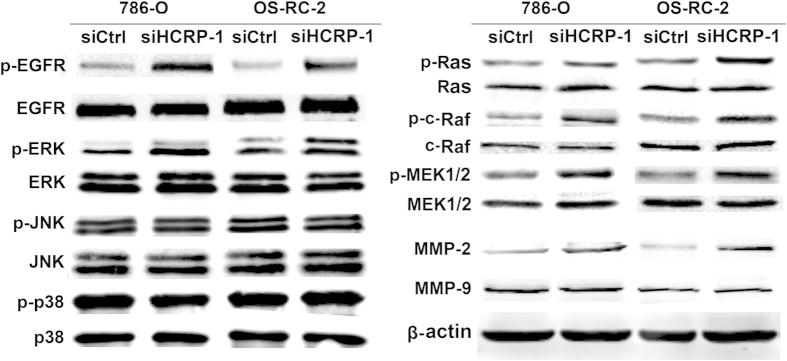
Loss of HCRP-1 induces MMP-2 expression, ERK activation and EGFR phosphorylation in RCC cells. Western blot analysis of the relative protein levels of MMP-2, MMP-9 and β-actin in silencing of HCRP-1 and control group for both 786-O and OS-RC-2 cell lines. Activities of EGFR and ERK were also determined by using Western blot analysis with antibodies specific for total and phosphorylated forms of EGFR and ERK. HCRP-1 knockdown activates the MAPK-ERK signaling pathway. Activities of JNK, p38 MAPK, Ras, c-Raf, and MEK1/2 were determined by using Western blot analysis with antibodies specific for total and phosphorylated forms of JNK, p38 MAPK, Ras, c-Raf, and MEK1/2 in silencing of HCRP-1 and control group for both 786-O and OS-RC-2 cell lines.

To investigate the mechanisms of HCRP-1 regulating migration and invasion, Western blot analysis was performed in RCC cells to detect the MMP levels, which are well-documented extracellular membrane-degrading enzymes associated with tumor migration and invasiveness. Our data showed that the MMP-2 protein, not the MMP-9, was dramatically induced in 786-O and OS-RC-2 cells transfected with HCRP-1 siRNA. Because previous studies have shown that the MAPK pathway is critical for the activation of MMP-2, we asked if HCRP-1 plays a role in regulating the MAPK pathway. We first examined the levels of phosphorylated (active) forms of MAPK family members (ERK1/2, JNK, and p38 MAPK) in 786-O and OS-RC-2 cells treated with HCRP-1 siRNA. As shown in Fig. 4, HCRP-1 depletion significantly increased ERK activation, but not the activation of JNK and p38 MAPK.

Further analysis of upstream components of the MAPK-ERK pathway showed that HCRP-1 depletion also induced the levels of phospho-ras, phospho-c-raf and phospho-MEK1/2 (Fig. 4), suggesting that HCRP-1 depletion plays a role in the activation of the Ras-Raf-MEK-ERK cascade.

As the role of HCRP-1 depletion in activation of the MAPK pathway, we postulated that HCRP-1 might regulate EGFR activation, which acts upstream of the MAPK-ERK pathway. Changes in EGFR activation were examined, and we found that EGFR phosphorylation (Tyr1173) was significantly elevated after HCRP-1 knockdown in RCC cell lines 786-O and OS-RC-2. On the other hand, HCRP-1 depletion induced EGFR phosphorylation in RCC cell lines, suggesting that HCRP-1 can negatively regulate EGFR activation (Fig. 4).

### Knockdown of HCRP-1 up-regulates MMP-2 expression through ERK signaling pathway

**Figure 5:**
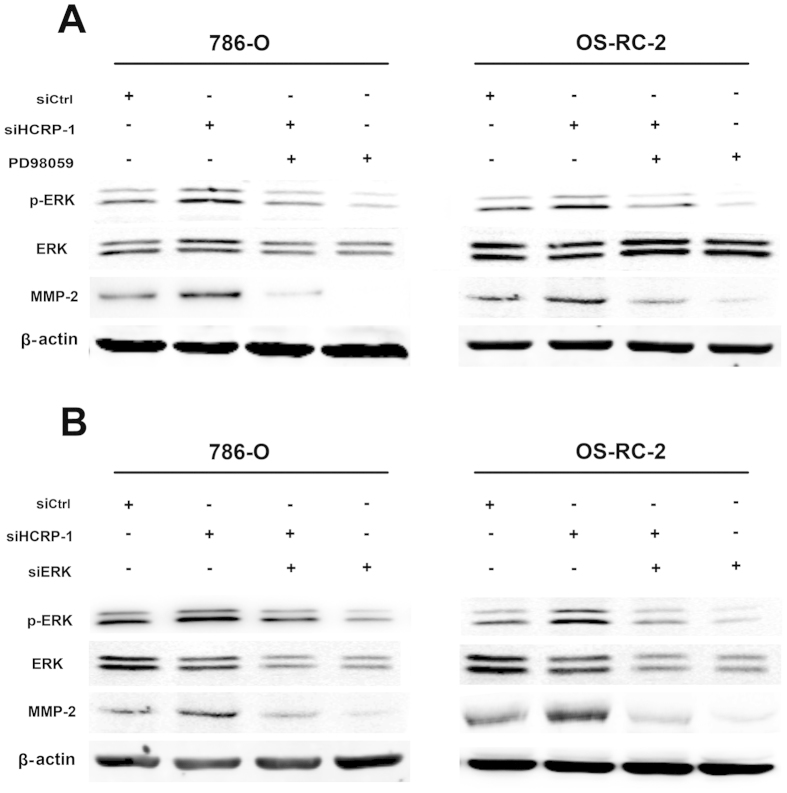
Knockdown of HCRP-1 up-regulates MMP-2 expression through MAPK-ERK signaling pathway. (**A**) 786-O and OS-RC-2 cells were pretreated with HCRP-1 siRNA and the MEK inhibitor, PD98059 (25 μmol/L). (**B**) 786-O and OS-RC-2 cells were pretreated with HCRP-1 siRNA and ERK siRNA. The whole cell lysates were analyzed for the protein levels of pERK and MMP-2. Levels of pERK1/2, ERK1/2, MMP-2, and β-actin were determined by using Western blot analysis.

**Figure 6:**
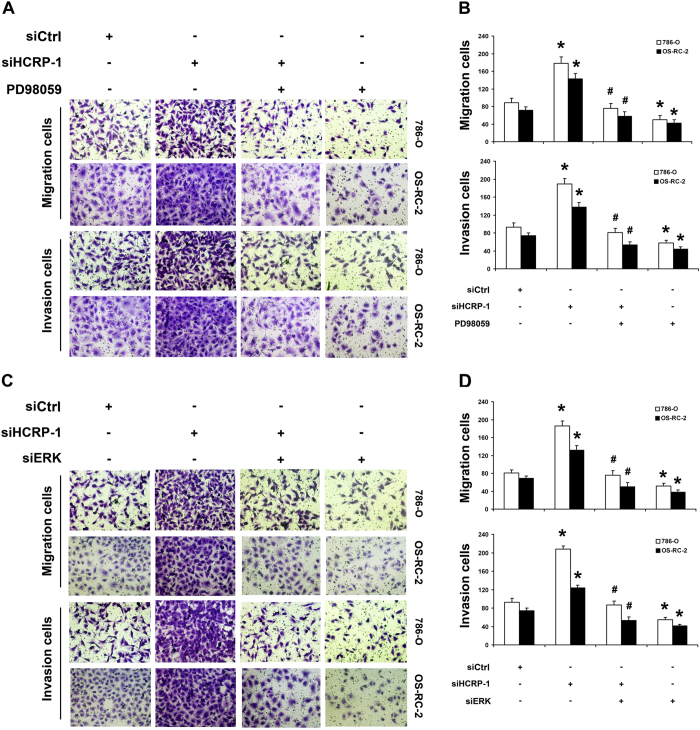
Knockdown of HCRP-1 induces cell migration and invasion through MAPK-ERK signaling pathway. (**A**–**D**) 786-O and OS-RC-2 cells were pretreated with HCRP-1 siRNA and the MEK inhibitor, PD98059 (25 μmol/L), or ERK siRNA. Cell migration and Matrigel cell invasion were evaluated after HCRP-1 siRNA treatment and treatment with PD98059 or ERK siRNA. Representative fields of migrating or invading cells on the membrane (magnification, ×200). The data are presented as mean ± SD for triplicate determinations. *P < 0.05 versus siCtrl group; ^#^*P* < 0.05 versus siHCRP-1 group.

Next, we asked whether ERK phosphorylation mediates MMP-2 expression which was induced by knockdown of HCRP-1. The MEK inhibitor, PD98059 (25 μmol/L) was used to inhibit ERK activation. As shown in Fig. 5A,B, PD98059 significantly inhibited ERK activation and expression of MMP-2 in HCRP-1-knockdown cells. Subsequently, we also found that migration and invasion abilities were suppressed by treatment of 786-O and OS-RC-2 cells with PD98059 (Fig. 6A,B).

Furthermore, we examined the effect of HCRP-1 on MMP-2 up-regulation in HCRP-1-deficient cells transfected with siRNA for ERK1/2. As shown in Fig. 5A,B, the expression of MMP-2 was suppressed and the ability to migrate or invade was decreased in RCC cells transfected with siRNA for ERK1/2. Obviously, the results were similar to the treatment of PD98059 (Fig. 6C,D). These findings collectively suggest that HCRP-1-knockdown leads to up-regulation of MMP-2 by activation of MAPK-ERK signaling pathway, leading to the ability of migration and invasion in 786-O and OS-RC-2 cells.

### Phosphorylation of EGFR is involved in the activation ERK signaling induced by depletion of HCRP-1

**Figure 7:**
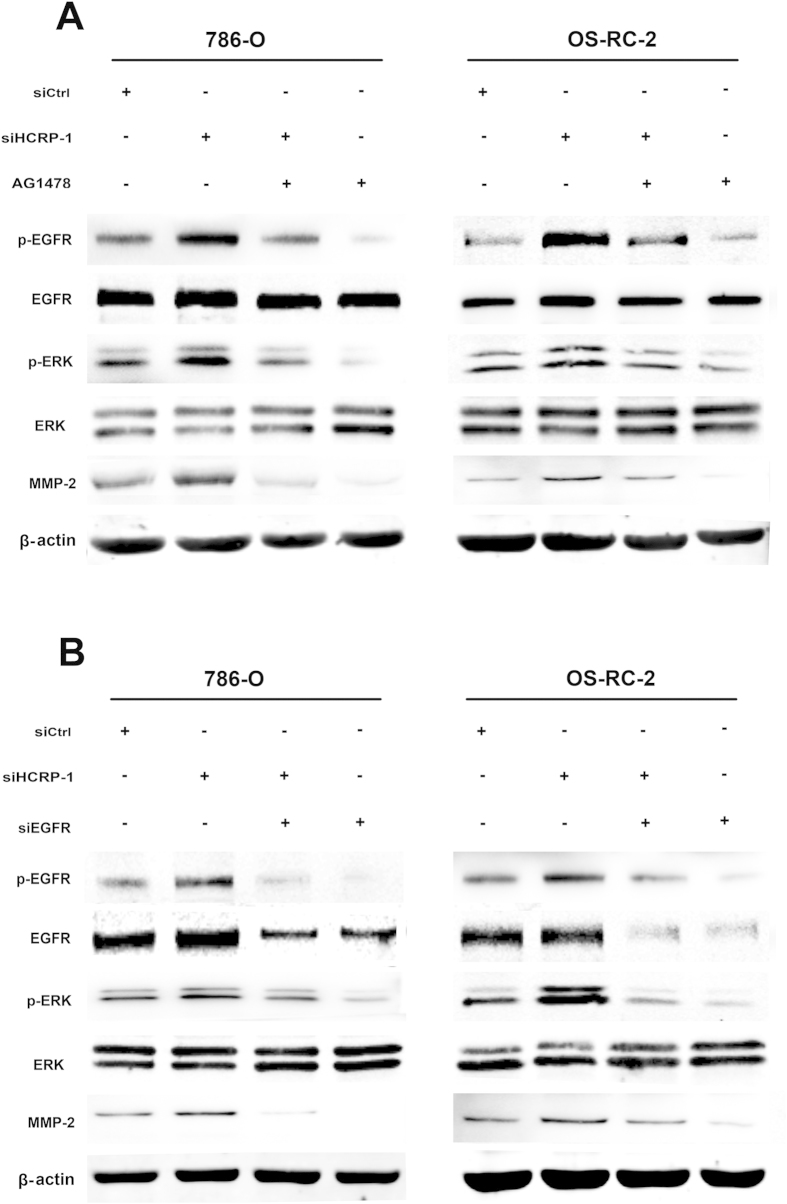
Inhibition of EGFR activation blocked HCRP-1-induced cell migration, invasion and MMP-2 expression. (**A**) 786-O and OS-RC-2 cells were pretreated with HCRP-1 siRNA and the EGFR inhibitor, AG1478 (1 nmol/L). (**B**) 786-O and OS-RC-2 cells were pretreated with HCRP-1 siRNA and EGFR siRNA. The whole cell lysates were analyzed for the protein levels of pEGFR and MMP-2. Levels of pEGFR, EGFR, MMP-2, and β-actin were determined by using Western blot analysis.

**Figure 8:**
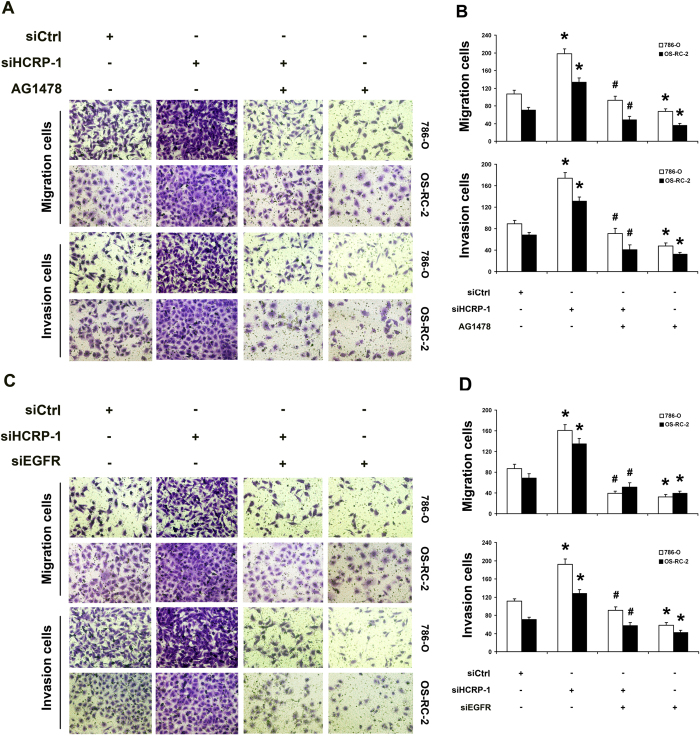
Inhibition of EGFR activation blocked HCRP-1-induced cell migration and invasion. (**A**–**D**) 786-O and OS-RC-2 cells were pretreated with HCRP-1 siRNA and the EGFR inhibitor, AG1478 (1 nmol/L), or EGFR siRNA. Cell migration and Matrigel cell invasion were evaluated after HCRP-1 siRNA treatment and treatment with AG1478 or EGFR siRNA. Representative fields of migrating or invading cells on the membrane (magnification, ×200). The data are presented as mean ± SD for triplicate determinations. *P < 0.05 versus siCtrl group; ^#^*P* < 0.05 versus siHCRP-1 group.

Furthermore, we investigated the role of EGFR phosphorylation in the activation ERK signaling induced by depletion of HCRP-1. To examine whether HCRP-1 mediated up-regulation of the ERK pathway is EGFR dependent, RCC cells 786-O and OS-RC-2 transfected with siRNA for HCRP-1 were treated with the EGFR inhibitor, AG1478 (1 nmol/L). As shown in Fig. 7A,B, AG1478 treatment inhibited EGFR phosphorylation, blocked HCRP-1-mediated up-regulation of phospho-ERK and MMP-2 expression. Cell migration and invasion abilities were also suppressed by treatment RCC cells with AG1478 (Fig. 8A,B). To further validate the role of EGFR phosphorylation in HCRP-1 mediated ERK and MMP-2 up-regulation, we used siRNA to deplete EGFR expression in 786-O and OS-RC-2 cells. As shown in Fig. 7A,B, the effect of siHCRP-1 on phospho-ERK and MMP-2 up-regulation were significantly blocked in EGFR-depleted RCC cell lines, and the ability to migrate or invade was also decreased in RCC cells transfected with siRNA for EGFR (Fig. 8C,D). These results were consistent with the group when treatment of AG1478. These data suggest that phosphorylation of EGFR is involved in the activation ERK signaling induced by depletion of HCRP-1.

## Discussion

There has been evidence indicating an important role for HCRP-1 in cancer development in some malignancies, such as ovarian cancer, HCC and breast cancer. In HCC, low HCRP-1 mRNA expression was independently associated with shorter disease-free survival^13^. Functional report showed that over-expression of HCRP-1 in the HCC cell lines significantly inhibited cell growth *in vitro*, whereas siRNA mediated knockdown of HCRP-1 in the HCC cell line resulted in increased cellular proliferation^13^. Besides in HCC, it has been clearly reported that reduced expression of HCRP-1 was observed in ovarian cancer, and loss of HCRP-1 drove invasive potential of cancer cells^14^. In addition, patients suffered from OOSCC were significantly associated with decreased overall survival or shorter disease-free survival only in tumors with low or missing HCRP-1 expression^15^. However, little is known about its expression pattern in RCC and its function in cancer progression.

In this study, we first identified decreased expression of HCRP-1 protein in RCC tissues. Our results demonstrated that HCRP-1 expression was decreased in RCC tissues compared with tumor adjacent normal renal tissues. In addition, we investigated the association between HCRP-1 expression and clinicopathological characteristics. Our data showed that decreased expression of HCRP-1 occurred in most of RCCs, and loss of expression was significantly correlated with histological grade, TNM stage and pT status. Furthermore, univariate and multivariate Cox proportional hazards regression analysis showed that loss of HCRP-1 expression was associated with poor outcome in terms of either overall survival or disease-specific survival. Collectively, these observations suggest that HCRP-1 is an important prognostic factor in RCC and may play a potential role in RCC metastasis.

Tumor cell migration and invasion are essential steps in the process of metastasis. To address the issue whether HCRP-1 can function in cancer metastasis of RCC, we used siRNA to knock down endogenous HCRP-1 in 786-O and OS-RC-2 cells, and observed that HCRP-1 depletion reduced cancer cell migration and invasion. MMPs, a family of zinc dependent endopeptidase, are significantly with cancer cells abilities of migration and invasion by degrading components of the basement membrane and extracellular matrix (ECM)^19^. The up-regulation of MMP-2 and MMP-9 is associated with poor prognosis in patients with RCC. Inhibition of MMP-2 and MMP-9 activity can suppress cancer metastasis^20^. We examined the levels of MMP-2 and MMP-9, our data demonstrated that knockdown of HCRP-1 may promote cancer cell migration and invasion through increased expression of MMP-2 but not the MMP-9. Herein, we report, for the first time to our knowledge that silence of HCRP-1 may contribute to the metastatic dissemination of RCC cells by promoting the expression of metastasis-related MMP-2, which supports the role of HCRP-1 on cell migration and invasion. However, the mechanism of up-regulation of MMP-2 by HCRP-1 and its signaling pathway to regulate RCC cells metastasis should remain to be explored.

In order to fully elucidate the mechanisms involved in HCRP-1-induced RCC cell migration and invasion, we further investigated the signaling pathways of MMP-2 associated with cellular invasion. It is well known that the MAPK signaling pathway regulates cancer cell invasion. The MAPK pathway targets the activating protein-1 family of transcription factors, including c-Fos and c-Jun family members, which have been known to activate MMP transcription^21^,^22^,^23^. Therefore, we focused on the MAPK pathways based on previous studies which showed that these pathways are involved in induced migration and invasion^24^. Our data demonstrated that only phosphorylation of MEK1/2 and ERK was detected in the induced migration and invasion of HCRP-1 depletion cells. Additionally, in support of this, pretreatment with PD98059, inhibitor of MEK1/2, and siRNA for ERK abrogated the activation of ERK, and thus reverses the siHCRP-1-induced MMP-2 expression and migration, invasion of 786-O and OS-RC-2 cells. The results suggested that activation of MEK-ERK signaling pathway is essential for the induced invasion of knockdown of HCRP-1 in RCC cells.

As a member of the ESCRT-I complex, HCRP-1 is involved in binding and sorting of ubiquitinated transmembrane proteins into MVBs and the consequent degradation with lysosomes^22^. It was reported that depletion of HCRP-1 strongly retards the degradation of EGFR^22^. In addition, Wittinger demonstrated that acquired resistance to cetuximab is linked to the accumulation of activated EGFR in the cytoplasm because of HCRP-1-related defects in receptor degradation, and HCRP-1-negative interfered EGFR signal transduction result in an increase in ERK1/2 phosphorylation. A previous report showed the link between activation of EGFR and Raf-MEK-ERK signaling^25^,^26^ and MMP production^27^. Recently, an oncogenic mechanism of EGFR-MEK-ERK-MMP was reported, which contributed to lung cancer invasion^28^. Given the potential role of HCRP-1 in EGFR signaling, we speculated that activation of endogenous EGFR signaling by under-expression of HCRP-1 could activate ERK1/2, which subsequently up-regulates MMP-2 expression. In support of this, the EGFR inhibitor AG1478 and siRNA for EGFR were used in our study and the data showed that up-regulation of MMP-2 expression and phosphorylation of ERK1/2 were reversed by the treatments in RCC cells. The abilities of cell migration and invasion were also decreased in RCC cells transfected with siRNAs for EGFR. These results demonstrated that loss of HCRP-1 can up-regulate EGFR phosphorylation, which subsequently drives downstream ERK-MMP-2 signaling.

**Figure 9:**
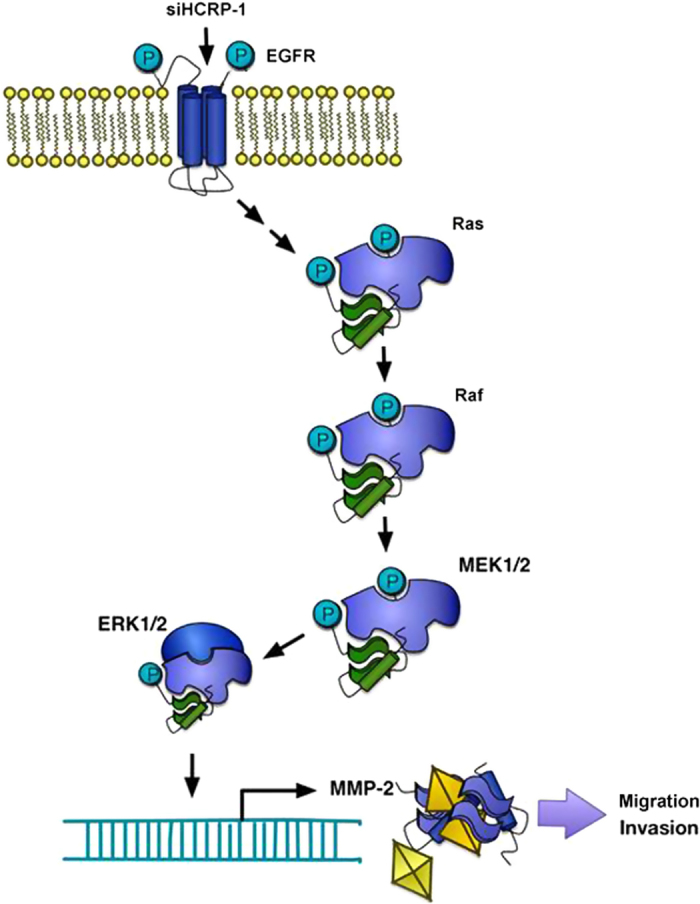
A proposed model of mechanisms involving in siHCRP-1-induced migration and invasion of human RCC cells.

In summary, our results showed that HCRP-1 is under-expressed in RCC and can influence migration and invasion by MMP-2 regulation, which is the result of EGFR-ERK pathway modulation, as is shown in Fig. 9. To our knowledge in this study, HCRP-1 is a novel tumor suppressor gene with an essential role in receptor tyrosine kinase degradation pathway, and could serve as a promising prognostic biomarker for RCC. Nonetheless, the promising role for HCRP-1 in migration and invasion of RCC cells merits further research, which may provide additional insight into its potential as a therapeutic target to decrease metastasis. We also propose further clinical relevance investigation for measuring HCRP-1 expression, to develop a new targeted-therapy to suppress RCC progression.

## Additional Information

**How to cite this article**: Chen, F. *et al.* HCRP-1 regulates cell migration and invasion via EGFR-ERK mediated up-regulation of MMP-2 with prognostic significance in human renal cell carcinoma. *Sci. Rep.* **5**, 13470; doi: 10.1038/srep13470 (2015).

## Supplementary Material

Supplementary Information

## Figures and Tables

**Table 1:** Patients characteristics and HCRP-1 expression.

Variables	HCRP-1 staining			
	Negative (%)	Positive (%)	Total	P*
All cases	38 (42.2)	52 (57.8)	90	
Age				
≤57 years	15 (39.5)	23 (60.5)	38	0.672
>57years	23 (44.2)	29 (55.8)	52	
Gender				
Male	26 (45.1)	25 (54.9)	51	0.084
Female	12 (38.5)	27 (61.5)	39	
Tumor size				
≤7 cm	25 (43.1)	33 (56.9)	58	0.665
>7 cm	13 (40.6)	19 (59.4)	32	
Grade				
G1	15 (35.6)	30 (64.4)	45	0.002
G2	10 (35.7)	18 (64.3)	28	
G3	12 (68.8)	4 (31.2)	16	
G4	1 (100.0)	0 (0.0)	1	
pT status				
pT_1_	25 (41.0)	36 (59.0)	61	0.001
pT_2_	8 (38.1)	13 (61.9)	21	
pT_3_	5 (62.5)	3 (37.5)	8	
TNM stage				
I	21 (35.0)	39 (65.0)	60	0.003
II	7 (43.8)	9 (56.2)	16	
III	7 (70.0)	3 (30.0)	10	
IV	3 (75.0)	1 (25.0)	4	

*P values are obtained from χ^2^ test.

**Table 2:** Univariate and multivariate survival analyses in RCC patients.

	Disease-specific survival				Overall survival			
	Univariate		Multivariate		Univariate		Multivariate	
	*P*-value	HR (95% CI)	*P*-value	HR (95% CI)	*P*-value	HR (95% CI)	*P*-value	HR (95% CI)
HCRP-1	0.001	3.847 (1.700–8.708)	0.024	0.379 (0.163–0.882)	0.000	4.003 (1.875–8.545)	0.020	0.384 (0.172–0.861)
Age	0.531	0.819 (0.439–1.529)	0.212	1.787 (0.719–4.444)	0.631	0.862 (0.470–1.580)	0.177	1.851 (0.757–4.528)
Gender	0.431	0.775 (0.416–1.410)	0.343	1.504 (0.647–3.495)	0.392	0.766 (0.416–1.410)	0.479	1.341 (0.595–3.024)
Tumor size	0.758	0.833 (0.410–1.936)	0.140	1.986 (0.798–4.942)	0.630	0.833 (0.394–1.757)	0.091	2.145 (0.886–5.189)
Tumor grade	0.045	4.101 (1.012–16.62)	0.361	1.572 (0.595–4.157)	0.021	3.444 (1.204–9.827)	0.265	1.695 (0.671–4.282)
TNM stage	0.022	3.441 (1.201–9.816)	0.018	2.981 (1.202–7.397)	0.019	4.295 (1.270–14.53)	0.024	2.767 (1.146–6.680)

Variables were analysed as follows: HCRP-1, negative vs positive; age, ≤57 years vs >57 years; tumor size, (≤7 cm vs >7 cm; tumor grade: G1-G2 vs G3-G4; TNM stage: I- II vs III- IV; pT status: I vs II-III.CI: confidence interval. HR: hazard ratio.

## References

[b1] Jemal A., Siegel R., Xu J. & Ward E. Cancer statistics, 2010. CA Cancer J. Clin. 60, 277–300 (2010). 20610543 10.3322/caac.20073

[b2] Comperat E. & Camparo P. Histological classification of malignant renal tumours at a time of major diagnostic and therapeutic changes. Diagn. Interv. Imaging 93, 221–231 (2012). 22465787 10.1016/j.diii.2012.01.015

[b3] Zisman A. *et al.* Risk group assessment and clinical outcome algorithm to predict the natural history of patients with surgically resected renal cell carcinoma. J. Clin. Oncol. 20, 4559–4566 (2002). 12454113 10.1200/JCO.2002.05.111

[b4] Cohen H. T. & McGovern F. J. Renal-cell carcinoma. N. Engl. J. Med. 353, 2477–2490 (2005). 16339096 10.1056/NEJMra043172

[b5] Hynes N. E., Horsch K., Olayioye M. A. & Badache A. The ErbB receptor tyrosine family as signal integrators. Endocr. Relat. Cancer 8, 151–159 (2001). 11566606 10.1677/erc.0.0080151

[b6] Hynes N. E. & Lane H. A. ERBB receptors and cancer: the complexity of targeted inhibitors. Nat. Rev. Cancer 5, 341–354 (2005). 15864276 10.1038/nrc1609

[b7] Holbro T., Civenni G. & Hynes N. E. The ErbB receptors and their role in cancer progression. Exp. Cell Res. 284, 99–110 (2003). 12648469 10.1016/s0014-4827(02)00099-x

[b8] Katzmann D. J., Odorizzi G. & Emr S. D. Receptor downregulation and multivesicular-body sorting. Nat. Rev. Mol. Cell Biol. 3, 893–905 (2002). 12461556 10.1038/nrm973

[b9] Kostelansky M. S. *et al.* Molecular architecture and functional model of the complete yeast ESCRT-I heterotetramer. Cell 129, 485–498 (2007). 17442384 10.1016/j.cell.2007.03.016 PMC2065850

[b10] Bache K. G. *et al.* The growth-regulatory protein HCRP1/hVps37A is a subunit of mammalian ESCRT-I and mediates receptor down-regulation. Mol. Biol. Cell 15, 4337–4346 (2004). 15240819 10.1091/mbc.E04-03-0250 PMC515363

[b11] Xu Z., Liang L., Wang H., Li T. & Zhao M. HCRP1, a novel gene that is downregulated in hepatocellular carcinoma, encodes a growth-inhibitory protein. Biochem. Biophys. Res. Commun. 311, 1057–1066 (2003). 14623289 10.1016/j.bbrc.2003.10.109

[b12] Zhang Q. *et al.* Aberrant methylation of the 8p22 tumor suppressor gene DLC1 in renal cell carcinoma. Cancer. Lett. 249, 220–226 (2007). 17029774 10.1016/j.canlet.2006.08.019

[b13] Lai M. W. *et al.* Expression of the HCRP1 mRNA in HCC as an independent predictor of disease-free survival after surgical resection. Hepatol. Res. 39, 164–176 (2009). 19208037 10.1111/j.1872-034X.2008.00413.x

[b14] Wittinger M. *et al.* hVps37A Status affects prognosis and cetuximab sensitivity in ovarian cancer. Clin. Cancer Res. 17, 7816–7827 (2011). 22016507 10.1158/1078-0432.CCR-11-0408

[b15] Perisanidis C. *et al.* HCRP1 expression status is a significant prognostic marker in oral and oropharyngeal cancer. Oral Dis. 19, 206–211 (2013). 22891969 10.1111/j.1601-0825.2012.01972.x

[b16] Veeratterapillay R. *et al.* Accuracy of the revised 2010 TNM classification in predicting the prognosis of patients treated for renal cell cancer in the north east of England. J. Clin. Pathol. 65, 367–371 (2012). 22287690 10.1136/jclinpath-2011-200468

[b17] Chen F. *et al.* RUNX3 suppresses migration, invasion and angiogenesis of human renal cell carcinoma. PLoS One 8, e56241 (2013). 23457532 10.1371/journal.pone.0056241 PMC3572981

[b18] Chen F. *et al.* Role of RUNX3 in suppressing metastasis and angiogenesis of human prostate cancer. PLoS One 9, e86917 (2014). 24475196 10.1371/journal.pone.0086917 PMC3901713

[b19] Deryugina E. I. & Quigley J. P. Matrix metalloproteinases and tumor metastasis. Cancer Metastasis Rev. 25, 9–34 (2006). 16680569 10.1007/s10555-006-7886-9

[b20] Roy R. *et al.* Tumor-specific urinary matrix metalloproteinase fingerprinting: identification of high molecular weight urinary matrix metalloproteinase species. Clin. Cancer Res. 14, 6610–6617 (2008). 18927302 10.1158/1078-0432.CCR-08-1136 PMC2879331

[b21] Chakraborti S., Mandal M., Das S., Mandal A. & Chakraborti T. Regulation of matrix metalloproteinases: an overview. Mol. Cell Biochem. 253, 269–285 (2003). 14619979 10.1023/a:1026028303196

[b22] Cho H. J. *et al.* Ascofuranone suppresses PMA-mediated matrix metalloproteinase-9 gene activation through the Ras/Raf/MEK/ERK- and Ap1-dependent mechanisms. Carcinogenesis. 28, 1104–1110 (2007). 17114644 10.1093/carcin/bgl217

[b23] Yen J. H., Kocieda V. P., Jing H. & Ganea D. Prostaglandin E2 induces matrix metalloproteinase 9 expression in dendritic cells through two independent signaling pathways leading to activator protein 1 (AP-1) activation. J. Biol. Chem. 286, 38913–38923 (2011). 21940623 10.1074/jbc.M111.252932 PMC3234716

[b24] Yang M. *et al.* NAIF1 inhibits gastric cancer cells migration and invasion via the MAPK pathways. J. Cancer Res. Clin. Oncol. 141, 1037–1047 (2014). 25432142 10.1007/s00432-014-1865-2 PMC11823667

[b25] Xiao L. J. *et al.* ADAM17 targets MMP-2 and MMP-9 via EGFR-MEK-ERK pathway activation to promote prostate cancer cell invasion. Int. J. Oncol. 40, 1714–1724 (2012). 22200661 10.3892/ijo.2011.1320

[b26] Ghosh S., Strum J. C., Sciorra V. A., Daniel L. & Bell R. M. Raf-1 kinase possesses distinct binding domains for phosphatidylserine and phosphatidic acid. Phosphatidic acid regulates the translocation of Raf-1 in 12-O-tetradecanoylphorbol-13-acetate-stimulated Madin-Darby canine kidney cells. J. Biol. Chem. 271, 8472–8480 (1996). 8626548 10.1074/jbc.271.14.8472

[b27] Kajanne R. *et al.* EGF-R regulates MMP function in fibroblasts through MAPK and AP-1 pathways. J. Cell. Physiol. 212, 489–497 (2007). 17348021 10.1002/jcp.21041

[b28] Dong Q. Z. *et al.* Derlin-1 is overexpressed in non-small cell lung cancer and promotes cancer cell invasion via EGFR-ERK-mediated up-regulation of MMP-2 and MMP-9. Am. J. Pathol. 182, 954–964 (2013). 23306155 10.1016/j.ajpath.2012.11.019

