# Corrigendum: HCRP-1 regulates cell migration and invasion via EGFR-ERK mediated up-regulation of MMP-2 with prognostic significance in human renal cell carcinoma

**DOI:** 10.1038/srep19412

**Published:** 2016-01-27

**Authors:** Feifei Chen, Junpeng Deng, Xin Liu, Wang Li, Junnian Zheng

Scientific Reports 5: Article number: 13470; 10.1038/srep13470published online 08252015; updated: 01272016

There is an error in the Acknowledgments section of this Article.

“This project is supported by grants from the National Natural Science Foundation of China (No. 81472663).“

should read:

“This project is supported by grants from the National Natural Science Foundation of China (No. 81302207).

