# Supplementary material for: HCRP-1 regulates cell migration and invasion via EGFR-ERK mediated up-regulation of MMP-2 with prognostic significance in human renal cell carcinoma
Source: Sci Rep. 2015 Aug 25;5:13470. doi: 10.1038/srep13470 (PMC4548257; doi:10.1038/srep13470)
Supplement: Supplementary Information [file srep13470-s1.doc]

**HCRP-1 regulates cell migration and invasion via EGFR-ERK mediated up-regulation of MMP-2 with prognostic significance in human renal cell carcinoma**

Feifei Chen^1^, Junpeng Deng^1,3^, Xin Liu^1^, Wang Li^2^, Junnian Zheng^1,2^

Feifei Chen and Junpeng Deng contributed equally to this paper

1, Jiangsu Center for the Collaboration and Innovation of Cancer Biotherapy, Cancer Institute, Xuzhou Medical College, Xuzhou, Jiangsu, China; 2, The Affiliated Hospital of Xuzhou Medical College, Xuzhou, Jiangsu, China; 3, Department of Urology, Suzhou Municipal Hospital, Suzhou, China.

Name: **Junnian Zheng**

Address: Jiangsu Center for the Collaboration and Innovation of Cancer Biotherapy, Cancer Institute, Xuzhou Medical College, 84 West Huai-hai Road, Xuzhou, Jiangsu, P.R.China

Tel: +86-0516-85802233;

^*^ E-mail: [jnzheng@xzmc.edu.cn](mailto:jnzheng@xzmc.edu.cn)

Supplementary materials: Human renal rubular epichelial cell line HK-2 was obtained from the Shanghai Institute of Biochemistry and Cell Biology, Chinese Academy of Sciences (Shanghai, China). HK-2 was cultured in Keratinocyte Serum Free Medium (K-SFM) supplemented with 10% fetal calf serum (Invitrogen, Shanghai, China). Rabbit monoclonal antibodies to VHL, HIF1α and HIF1β were purchased from Cell Signaling Technology (Beverly, MA).

Supplementary Figure S1: VHL expression was low but HIF1α and HIF1β expression was high in RCC cell lines. Western blot analysis of the relative protein levels of MMP-2, MMP-9 and β-actin in RCC cell lines 786-O and OS-RC-2 and renal rubular epichelial cell line HK-2.


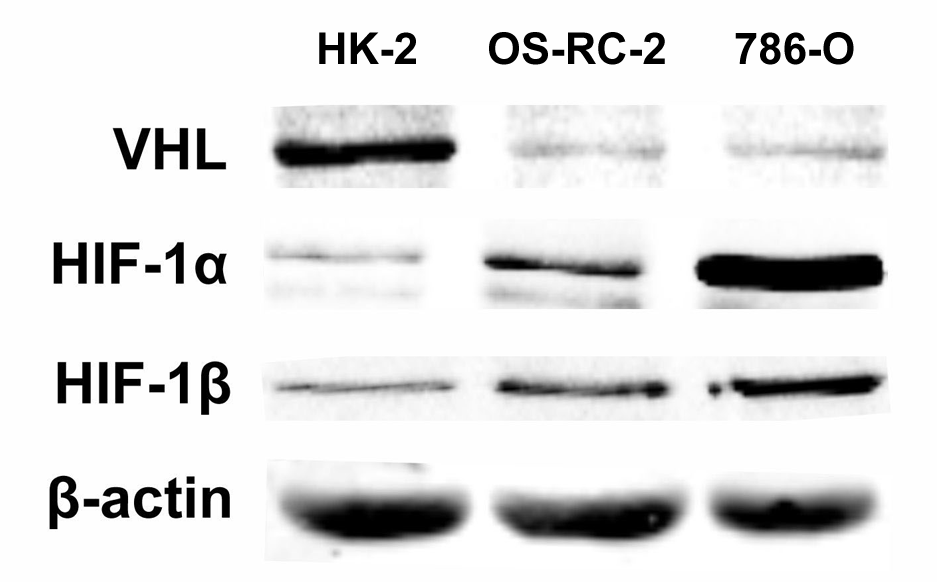


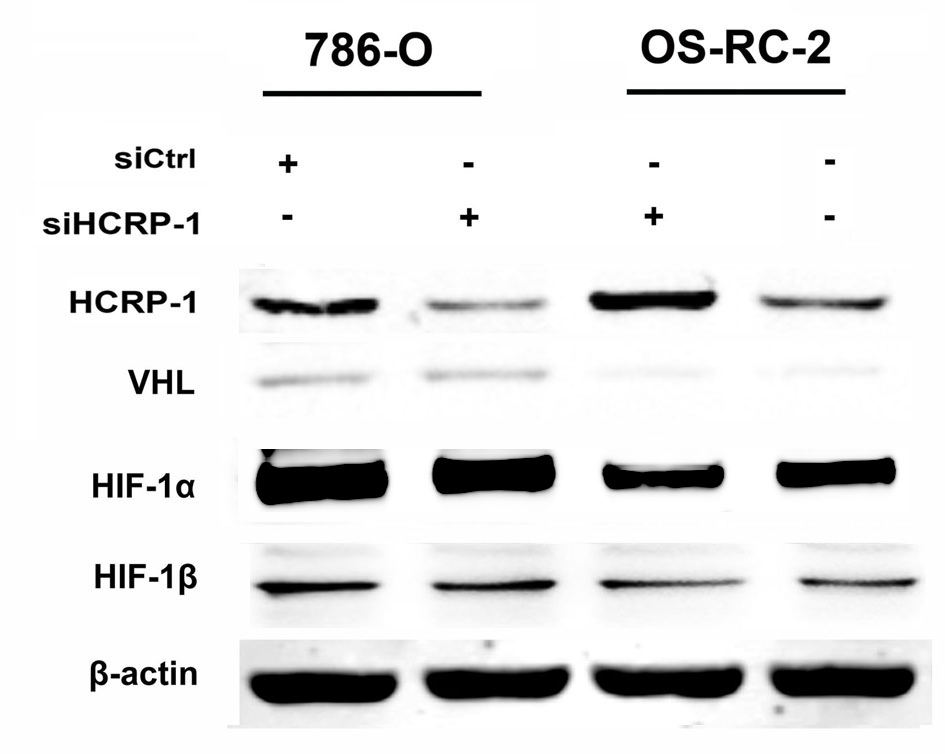
Supplementary Figure S2: si-HCRP-1 didn’t affect the expression of VHL, HIF1α and HIF1β. Western blot analysis of the relative protein levels of HCRP-1, VHL, HIF1α, HIF1β and β-actin after HCRP-1 knockdown for both 786-O and OS-RC-2 cell lines.
